# In Search of Herbal Anti-SARS-Cov2 Compounds

**DOI:** 10.3389/fpls.2020.589998

**Published:** 2020-11-16

**Authors:** Tatiana Matveeva, Galina Khafizova, Sofia Sokornova

**Affiliations:** ^1^Department of Genetics and Biotechnology, St. Petersburg State University, St. Petersburg, Russia; ^2^Department of Toxicology and Biotechnology, All-Russian Institute of Plant Protection, St. Petersburg, Russia

**Keywords:** genetic engineering, cheminformatics, secondary metabolites, antiviral drugs, coronavirus, medicinal plants (herbal drugs), biochemical methods

## Abstract

On March 11, 2020, the World Health Organization (WHO) announced that the spread of the new coronavirus had reached the stage of a pandemic. To date (23.10.2020), there are more than 40 million confirmed cases of the disease in the world, at the same time there is still no effective treatment for the disease. For management and treatment of SARS-Cov2, the development of an antiviral drug is needed. Since the representatives of all human cultures have used medicinal plants to treat viral diseases throughout their history, plants can be considered as sources of new antiviral drug compounds against emerging viruses. The huge metabolic potential of plants allows us to expect discovery of plant compounds for the prevention and treatment of coronavirus infection. This idea is supported by number of papers on the anti-SARS-Cov2 activity of plant extracts and specific compounds in the experiments *in silico*, *in vitro*, and *in vivo*. Here, we summarize information on methods and approaches aimed to search for anti-SARS-Cov2 compounds including cheminformatics, bioinformatics, genetic engineering of viral targets, interacting with drugs, biochemical approaches etc. Our mini-review may be useful for better planning future experiments (including rapid methods for screening compounds for antiviral activity, the initial assessment of the antiviral potential of various plant species in relation to certain pathogens, etc.) and giving a hand to those who are making first steps in this field.

## Introduction

A novel coronavirus strain causing fatal respiratory syndrome was reported in late 2019. In January 2020, it was revealed that it belongs to the beta-coronaviruses, sharing similarity to SARS-coronaviruses, and that its spike protein interacts strongly with the human angiotensin-converting enzyme 2 (ACE2) receptor (Dhama et al., 2020; Xu et al., 2020). On March 11, the World Health Organization (WHO, 2020) announced that the spread of the new coronavirus had reached a pandemic stage. To date (23.10.2020), there are more than 40 million confirmed cases of the disease in the world, while there is still no effective treatment for the disease. In this regard, the search for cures for this disease is undoubtedly relevant and significant. Plant-based medicine is attracting a lot of attention today, since medicinal plants are enriched with variety of secondary metabolites including those with antiviral properties (Gurib-Fakim, 2006; Adedeji and Sarafianos, 2014; Dhama et al., 2018; Divya et al., 2020; Vellingiri et al., 2020). About, a third of FDA-approved drugs over the past 20 years are based on natural products or their derivatives (Carter, 2011).

Natural products can offer safe and inexpensive platforms for discovery of efficient and novel agents for treatment of SARS-CoV-2 with minimizing side effects (Ghildiyal et al., 2020; Huang et al., 2020; Mani et al., 2020). Although structures for 200,000 natural products are known, only 15% of the estimated 350,000 plant species have been investigated for their chemical constituents (Cragg and Newman, 2013). Thus, plants hold great potential as a material for drug development.

## Potential Targets for Anti-SARS-Cov2 Drug Design

Coronaviruses are single-stranded (+) RNA viruses, having four structural proteins: spike (S), envelope (E), membrane (M), and nucleocapsid (N) proteins. The other 25 nonstructural proteins (NSPs) regulate assembling of copies of virus particles and their passing through the host’s immune system (Xu et al., 2020).

Inside the host cell, two polyproteins, pp1a and pp1ab, are directly translated from the viral RNA and then cleaved by two viral proteases, main protease (M^pro^, also called 3-chymotrypsin-like cysteine protease, or 3CL^pro^) and papain-like protease (PL^pro^). PL^pro^ cleaves junctions of NSP 1, 2, and 3 while M^pro^ cleaves polyprotein at 11 distinct sites downstream from the NSP4 to generate various NSPs that are important for viral replication (Perlman and Netland, 2009). The M^pro^ is one of the best-characterized drug targets among the coronaviruses (Anand et al., 2003; Hilgenfeld, 2014). Since no human proteases with similar cleavage specificity are known, the inhibitors of M^pro^ are unlikely to be toxic (Zhang et al., 2020b). The PL^pro^, is also attractive antiviral drug target, because it affects not only coronaviral replication, but also has the additional function of deubiquitination of host cell proteins and ISG15 removal, finally leading to the immune suppression of host cells (Báez-Santos et al., 2015; Lin et al., 2018; Clemente et al., 2020).

The viral S protein is essential for viral attachment, fusion, and entry. It uses host angiotensin-converting enzyme 2 (ACE2) as a receptor to get into the host cells. Since, the structure of S protein of SARS-CoV-2 is revealed (Coutard et al., 2020; Huang et al., 2020; Wrapp et al., 2020) and receptor-binding domain was identified, it can serve as a target for development of inhibitors of S protein and ACE2 interaction (Tai et al., 2020).

Approaches to search for substances interacting with these targets will be described below together with successful examples of their application.

## Drug Search Strategies in Phytomedicine

Previously, new drugs were discovered mostly by a “trial and error” approach (Butcher et al., 2004). As methods and knowledge in chemistry advance, researchers began to purify the active compounds in herbal extracts known to have medicinal properties and determine their structures (Drews, 2000). The emergence of genomics and proteomics and the development of bioinformatics and cheminformatics made a breakthrough into the drug design, bringing the concept and techniques for large-scale screening. Newly emerging diseases require faster pace of drug development. Worldwide transmission of COVID-19 (van Dorp et al., 2020) and high infectivity (Zhang and Holmes, 2020) of the virus demands rapid development of suitable drugs.

In phytomedicine, there are several schemes used for this purpose. Let us focus on each of them.

### Classical Approach

Classical approach represents either the screening based on previously purified natural compounds or on activity-guided screening of crude extract mixture for active compounds. Further, active compounds are purified by activity-guided purification (Szajdak, 2016). This way of identification of antiviral compounds was implemented by Li et al. (2005). More than 200 Chinese medicinal herb extracts were screened for anti-SARS-CoV activities by assay for virus-induced cytopathic effect. The crude extracts of *Lycoris radiata*, *Artemisia annua*, *Pyrrosia lingua*, and *Lindera aggregate* showed antiviral activity. The ethanolic extract of *L. radiata* was the most active. The majority of bioactive components of *L. radiata* belong to alkaloids that were confirmed by reversed phase high performance liquid chromatography (LC). Finally, lycorine was identified by LC-MS/MS as an effective anti-SARS-CoV component. Now, anti-SARS-CoV2 effect of the lycorine is also shown (Murck, 2020).

Often activity-guided screening indicates that plant fractions have higher antiviral activity than pure substances, because medicinal plants usually contain several biologically active compounds. For example, *Kickxia elatine*, contain flavonoid pectolinarin (Yuldashev et al., 1996) and iridoid glycosides with anti-inflammatory and antiviral activities (Handjieva et al., 1995), and therefore, provide some synergetic. Besides, some compounds are able to prevent disease by various means. For example, pectolinarin efficiently blocks the enzymatic activity of M^pro^ and PL^pro^ (Jo et al., 2020) and demonstrates anti-inflammatory activities (Ho et al., 2020).

### Structure-Activity-Relationship Approach

Structure-activity-relationship is an approach for finding the relationships between the chemical structure (structural-related properties) and the biological activity of studied compounds. The need to streamline the drug development process has spawned the development of such strategies as “rational drug design,” where large-scale screening can be done through a database of potential candidate molecules to find those of interest (https://www.ebi.ac.uk/chembl, https://pubchem.ncbi.nlm.nih.gov/, Santos et al., 2016; Mumtaz et al., 2017). Medicinal plants contain wide range of secondary metabolites of different activity spectrums. Some of them are known to have antiviral and other properties, for others only structural data is available. The properties of many plants-derived substances remain unclear. These unknown properties can be predicted by computer modeling methods.

Topological indices are able to predict different activities and physicochemical characteristics such as boiling point, entropy, enthalpy, etc. Similarly, based on the topological properties of some chemical structures with antiviral activity, (for example remdesivir, chloroquine, hydroxychloroquine, and theaflavine), the antiviral activity of the new compounds can be predicted (Mondal et al., 2020).

Antiviral features of compounds can be determined by matching them as a ligand to known targets using molecular dynamics and docking simulations (Zhang et al., 2020a). Tallei et al. (2020) made a list of natural components with M^pro^-inhibitor effect; among them are hesperidin, morine, rhoifolin, pectolinarin, and nabiximols. Binding interaction and ligand affinity of these compounds to M^pro^ was the same as of nelfinavir, and even better than chloroquine and hydroxychloroquine sulfate, − recommended by the FDA as emergency anti-COVID-treatment anti-malarial drugs (Tallei et al., 2020). Quercetin-3-β-galactoside showed inhibitory activity against SARS-CoV M^pro^ *in silico*, *via* docking simulation, and also in enzymatic inhibition assays. Molecular modeling strongly suggested that the residue Q189 plays a key role, and it was confirmed by site-directed mutagenesis of the M^pro^ (Chen et al., 2006). Molecular docking analysis of M^pro^ and compounds of medicinal plants revealed such inhibiting substances as beta-eudesmol from *Lauris nobilis*, digitoxigenin from *Nerium oleander*, crocin from *Crocus sativus* (Aanouz et al., 2020), pavetannin-C1 and tenuifolin from cinnamon (Prasanth et al., 2020), catechins/polyphenols from green tea (Ghosh et al., 2020), withanoside V from *Withania somnifera* (Tripathi et al., 2020), and tinosponone from *Tinospora cordifolia* (Krupanidhi et al., 2020). Inhibitory effects of alkylated chalcones isolated from *Angelica keiskei* against the SARS-CoV proteases M^pro^ and PL^pro^ were found by Park et al. (2016).

Quercetin showed inhibitory activity against SARS-CoV PL^pro^, although Papyriflavonol A was the most effective inhibitor of PL^pro^ among those studied by Park et al. (2017). Virtual structure-based screening revealed that withanolide A, isocodonocarpine, and calonysterone bind to PL^pro^ (Alamri et al., 2020).

Molecular docking analysis applied on the binding positions with S protein indicated that cannabinoids along with epigallocatechin gallate, herbacetin, hesperidine, pectolinarin, curcumin, and withanoside X hold remarkable binding sites, which could support them to be excellent S protein inhibitors, preventing viral attachment to host cells (Chikhale et al., 2020; Jena et al., 2020; Tallei et al., 2020).

Unfortunately, molecular docking does not consider the effect of selected substances on other key points of the disease and the cumulative effect of several biologically active compounds. However, concept of large-scale screening can be applied to identify new candidates for further analysis, shortening the search process and making it more powerful (Chen et al., 2006; Theerawatanasirikul et al., 2020).

### The Data-Driven Approach

Repurposing of known drugs could significantly accelerate the deployment of novel therapies for COVID-19. The main difference of data-driven approach from the structure-activity-relationship approach is in use of databases of drugs, including phytochemical ones (https://www.drugbank.ca, http://drugcentral.org etc.). The identification of new areas of application for already known drugs saves time for testing their biosafety, but does not provide an opportunity to find fundamentally new substances that can be more effective than previously known drugs. The approach includes three steps:

1. Selection of drugs aimed to treat “Warm diseases,” “Pestilence,” or “Epidemic diseases.”
2. Identification of the active substances of plant origin, responsible for these pharmacology effects.
3. *In silico* assessment of the activities of selected drugs in relation to key proteins (Mpro, PLpro S protein, and ACE2) involved in the development of the disease.

According to this scheme, among the 96,606 formulations of Traditional Chinese Medicine, the 574 drugs were selected, and only 26 ones remained after the third stage of analysis. These biologicaly activity substances from the licorice (*Glycyrrhizae radix*) and skullcaps (*Scutellariae baicalensis*) could also interact with the targets involving in immune and inflammation diseases (Ren et al., 2020).

Similar approach based on the ReFRAME (Repurposing, Focused Rescue, and Accelerated Medchem) library was utilized by Riva et al. (2020).

### Immunomodulatory Effects of Herbs

As we can see from the examples mentioned above, extracts of many plants, and even specific compounds, often inhibit several SARS-CoV2 protein targets. Besides, identified compounds can possess a wide range of other pharmacological activities, including immunomodulatory effects. For example, Curcumin inhibits S protein (Jena et al., 2020) and also participates in regulation of immune and inflammatory response associated with coronavirus infections (Chen et al., 2020).

Moreover, plants often contain a number of compounds, with different activities that can complement each other. Thus, *Cirsium japonicum*, popular in traditional Chinese medicine, contains pectolinarin and pectolinarigenin, which have antiviral and immunomodulatory effects, respectively (Cheriet et al., 2020). Pectolinarigenin shows high suppressive immunomodulatory potency, including inhibitory activity of neutrophil phagocytes respiratory burst as well as T-cells proliferation (Erukainure et al., 2017).

*Withania somnifera*, a key plant of Ayurveda, contains compounds inhibiting M^pro^ and S protein, (Chikhale et al., 2020; Tripathi et al., 2020) and the extract of the plant provides anti-viral immunity by increasing interferon gamma responses and anti-inflammatory activities by decreasing the quantity of interleukin-1, interleukin-6 and Tumor necrosis factor related to COVID-19 (Niraj and Varsha, 2020).

Tinosponone from *Tinospora cordifolia* inhibits M^pro^ (Krupanidhi et al., 2020), and the aqueous extracts of the plant affects the cytokine production and activation of immune effector cells (Niraj and Varsha, 2020).

The immune response against coronavirus is vital to control and get rid of the infection. In the ideal situation, the antiviral drug should check the infection while the immune system prepares to destroy the last virus particles (Mukherjee, 2019) From other hand, maladjusted immune responses may lead to the immunopathology of the disease, resulting in impairment of pulmonary gas exchange (Dhama et al., 2020). Thus, a fine selection of herbal immunomodulators is required for the treatment of different stages of the diseases and different degrees of its severity.

## Genetic Engineering for Analysis of Anti-SARS-Cov2 Activity

Genetic engineering methods allow to optimize the evaluation of anti-SARS activity of compounds at different stages of research. First of all, they include obtaining of recombinant target proteins for searching for antiviral drugs.

The recombinant proteases M^pro^ and PL^pro^ can be easily expressed in *Escherichia coli* or other organism, purified by routine biochemical methods (Lin et al., 2004, 2005) and used for cell-free cleavage assay (Lin et al., 2005; Chou et al., 2008; Chen et al., 2009; Jo et al., 2020) or cell-based cleavage assay (Lin et al., 2005), as well as for study of x-ray structures of the unliganded SARS-CoV-2 proteins and their complexes with potential drugs (Zhang et al., 2020b).

Cell-free cleavage assay involves the use of a purified enzyme and a substrate, modified at the C and N terminus. Depending on the type of modification (protein tag or fluorescent group), further Enzyme Linked Immunosorbent Assay (ELISA) or protein based fluorogenic assay are used to assess cell-free proteolytic activity and its inhibition by different compounds (Kuo et al., 2004; Lin et al., 2005).

The cell-based cleavage assay does not require purification of the active protease, and represents closely the natural physiological state. Investigating the *Isatis indigotica* phenolic compounds as potential anti-SARS drugs, Lin et al. (2005) have made in-frame construction, containing the M^pro^, the substrate, and the luciferase, and transformed it into Vero cells. Since a more than 30 kDa protein fused at the N-terminus of the luciferase resulted in a dramatic decrease of luciferase activity, the detection of activity of luciferase was considered by authors as a measure for the cis-cleavage by M^pro^. Epigallocatechin gallate abundant in green tea (*Camellia sinensis*), inhibits the proteolytic activity of SARS-CoV M^pro^, expressed in *Pichia pastoris* (Nguyen et al., 2012).

In addition, approaches described above, demonstrated that tanshinones of *Salvia miltiorrhiza* (Park et al., 2012b), diarylheptanoids from *Alnus japonica* (Park et al., 2012a), and geranylated flavonoids from the *Paulownia tomentosa* tree (Cho et al., 2013) are inhibitors of SARS-CoV PL^pro^ activity. M^pro^ proteases of SARS-CoV and SARS-CoV-2 are inhibited by such compounds as herbacetin, rhoifolin, pectolinarin (Jo et al., 2020) hesperetin, sinigrin (Lin et al., 2005) quercetin-3-β-galactoside (Chen et al., 2006) etc.

Thus, at the initial stages, the assessment of the antiviral activity of drugs does not require the use of the virus itself. This makes it possible to significantly expand the number of laboratories where it is permissible to conduct such studies and select the best compounds for the next stages of testing.

Genetic engineering technologies are also applicable at the stage of evaluating the therapeutic effect of drugs using preclinical animal models. Several animal models, from mice to and non-human primates, have been shown to be susceptible to SARS-CoV infection. From one hand, primates are closest to humans; the clinical picture of their disease is almost the same as in humans. From other hand, they are much more expensive than mice. Therefore, drugs are initially tested on mice (Bevinakoppamath et al., 2020; Lutz et al., 2020). Unfortunately, due to structural differences in mouse ACE2 compared to human ACE2 proteins, the SARS coronaviruses exhibit poor tropism characteristics for mouse tissues. So, the wild-type lines are not optimal for studying infections of the newly discovered coronavirus. Several transgenic mice strains, carrying hACE2 under control of different promoters, including human cytokeratin 18 (K18) promoter, composite CAG promoter consisting of the cytomegalovirus immediate early enhancer, the chicken β-actin promoter and rabbit globulin splicing and polyadenylation sites, HFH4 lung ciliated epithelial cell-specific promoter, were developed in 2006–2016 to study SARS-CoV infection (McCray et al., 2007; Tseng et al., 2007; Menachery et al., 2016). It was shown that the hACE2 transgenic mice infected with a human SARS-CoV strain *via* intranasal inoculation demonstrate the symptoms observed in infected human patients. These hACE2 transgenic mice can provide significant findings to support the development of COVID-19 therapeutics (Lutz et al., 2020).

## Taxonomic Studies and Genome Sequencing Searching for Better Producers of Phytochemical Drugs

For a more efficient search for better plant producers of specific secondary metabolites and their combinations, it makes sense to use the concept of plant chemotaxonomy – a branch of the science of taxonomy, were plants are classified depending on the similarities and differences in the spectrum of their secondary metabolites. Since some chemicals can be found in nature only in organisms of certain genera, families, or orders, this can be used both for their classification and to search for certain metabolites in the related species (Zidorn, 2019).

Whole genome data in its turn led to insights into biosynthesis pathways (Denoeud et al., 2014). The list of species with sequenced genomes is growing, collaborations are emerging to join efforts and collect more data. The Medicinal Plant Genomic Resource (MPRG)^1^ is a domain-specific database created by consortium efforts to collect deep DNA sequencing, RNA sequencing, and metabolomics data. In general, genome sequencing is used to discover new candidates and to elucidate biosynthesis pathways to prepare data sets of new molecules for further analysis. One can predict the presence of a compound among plant metabolites if gene involved in the biosynthesis of the compound in question was discovered in the plant genome. For example, cardenolides discovered in the *Calotropis gigantea* genome were historically used to cure pneumonia and as an anti-inflammatory, anticancer, and antimalaria (Hoopes et al., 2018; Boone et al., 2020), and they also can be easily converted to mappicine ketone, an antiviral led compound (Das et al., 1998). Genes to biosynthesize curcumin were found in *Oryza sativa* genome (Katsuyama et al., 2007). Recently conducted chromosome-scale genome assembly of *I. indigotica* assisted to reveal new candidate genes for the biosynthesis of several groups of active compounds in this medicinal plant (Kang et al., 2020). Phenolic compounds of *I. indigotica* have already shown activity against the M^pro^ (Lin et al., 2005), and indole alkaloids in this plant demonstrated inhibition of HSV-2 reproduction (Sun et al., 2010). Besides, cultivars of the same species can differ in the amount of the medicinal compounds (Hisashi and Saito, 2013; Kajikawa et al., 2017). Complete genome data allow to identify gene clusters for secondary metabolism, which opens the way to metabolic engineering. For example, whole-genome sequencing can reveal huge number of “silent” gene clusters which can be learned to activate to run currently non-working biosynthetic pathways (Osbourn, 2010).

Thus, in the nearest future chemotaxonomy and genomic data will allow to improve the procedure for obtaining phytochemical drugs.

## Conclusion

In this short article, we have tried to present various methods of searching for plant compounds with anti-coronavirus activities, as well as approaches that make it possible to accelerate this search. Chemi-informatics methods provide opportunities for primary screening of large amounts of data to find candidate compounds. Genetic engineering methods make it possible to assess the interaction of candidate compounds with their targets in cell-free systems and in cell culture without resorting to the use of viruses, which significantly expands the list of laboratories for such research. Combining these approaches allows for more accurate study of targeting of potential drugs for subsequent trials. Acceleration of the preclinical phase of new drugs testing can be achieved through the use of transgenic animal models.

Analysis of pharmacological databases allows quickly selection of “candidates,” already having assessments of their biosafety and stability, but it is unlikely that a “breakthrough” drug will be obtained at the exit. However, these approaches buy time to treat people while longer studies are still pending.

The “hunt” for new substances makes it possible to find substances with new mechanisms of action, reveal new functional groups, etc. The most effective drugs may be among them. However, this path is the longest, since it will require the most complete study of its pharmacological properties, including toxicity, side effects, stability, etc. Therefore, rapid screening systems for target activities are extremely important.

The analysis of a mixture of compounds in plant extracts seems to us the most promising. The development of metabolomics methods based on a small amount of plant material allows to obtain data on the entire spectrum of substances in the sample under study, containing several compounds with antiviral activity.

In the future, our knowledge of the structure of plant genomes will allow to obtaining the most effective producers of anti-coronavirus compounds by metabolic engineering methods.

## Author Contributions

TM wrote the basic structure of the paper. GK and SS participated in writing and correcting the paper. All authors contributed to the article and approved the submitted version.

### Conflict of Interest

The authors declare that the research was conducted in the absence of any commercial or financial relationships that could be construed as a potential conflict of interest.

## Footnotes

^1^http://medicinalplantgenomics.msu.edu
